# Effect of Sodium-Glucose Cotransporter-2 (SGLT-2) Inhibitors on Chronic Kidney Disease Progression: A Systematic Review

**DOI:** 10.7759/cureus.115840

**Published:** 2026-09-05

**Authors:** Diana D Nenova, Yanko G Yankov, Gergana M Chausheva

**Affiliations:** 1 Department of Nephrology, Medical University "Prof. Dr. Paraskev Stoyanov", Varna, BGR; 2 Clinic of Nephrology and Dialysis, University Hospital "St. Marina", Varna, BGR; 3 Clinic of Maxillofacial Surgery, University Hospital "St. Marina", Varna, BGR; 4 Department of General and Operative Surgery, Medical University "Prof. Dr. Paraskev Stoyanov", Varna, BGR; 5 Department of Clinical Laboratory, Medical University "Prof. Dr. Paraskev Stoyanov", Varna, BGR; 6 Department of Central Clinical Laboratory, University Hospital "St. Marina", Varna, BGR

**Keywords:** acute kidney injury, chronic kidney disease, ckd progression, estimated glomerular filtration rate, kidney failure, kidney outcomes, nephroprotection, sglt-2 inhibitors, sodium-glucose cotransporter-2 inhibitors, systematic review

## Abstract

Chronic kidney disease (CKD) is a major global health concern characterized by progressive loss of kidney function and an increased risk of cardiovascular morbidity and mortality. Sodium-glucose cotransporter-2 (SGLT-2) inhibitors, initially developed as antihyperglycemic agents for the treatment of type 2 diabetes mellitus (T2DM), have subsequently demonstrated substantial renoprotective effects that extend beyond glycemic control. This systematic review aimed to evaluate current evidence regarding the effects of SGLT-2 inhibitors on CKD progression in adults with and without diabetes.

A comprehensive literature search was conducted in PubMed/MEDLINE, Scopus, and Google Scholar from database inception through October 2025. Eligible studies included randomized controlled trials (RCTs), systematic reviews and meta-analyses, and prospective or retrospective observational cohort studies evaluating kidney outcomes, including CKD progression, changes in estimated glomerular filtration rate (eGFR), doubling of serum creatinine, and progression to end-stage kidney disease (ESKD). The review was conducted in accordance with the Preferred Reporting Items for Systematic Reviews and Meta-Analyses (PRISMA) 2020 guidelines.

Thirty-two studies met the inclusion criteria, comprising 13 RCTs, eight systematic reviews and meta-analyses, and 11 observational cohort studies. Across randomized trials, SGLT-2 inhibitors consistently reduced CKD progression and major adverse kidney outcomes and slowed the decline in kidney function. Dedicated kidney outcome trials demonstrated substantial reductions in adverse kidney outcomes, while meta-analytic evidence confirmed preservation of kidney function across diverse CKD populations, including individuals without diabetes. Real-world observational studies further supported these findings, demonstrating slower eGFR decline and favorable kidney outcomes in routine clinical practice. SGLT-2 inhibitors were generally well tolerated, and meta-analytic evidence demonstrated a reduced risk of acute kidney injury among patients with CKD.

Collectively, the available evidence indicates that SGLT-2 inhibitors substantially slow CKD progression and reduce adverse kidney outcomes across diverse CKD populations, including patients with and without diabetes. Their consistent efficacy and favorable safety profile support their role as cornerstone nephroprotective agents in contemporary CKD management. Further research is warranted to clarify their long-term efficacy and safety in underrepresented populations, particularly patients with advanced CKD and non-albuminuric disease.

## Introduction and background

Chronic kidney disease (CKD) is a major global public health challenge, affecting a substantial proportion of the adult population and imposing a considerable burden through increased cardiovascular morbidity, premature mortality, and healthcare expenditure [1]. The disease is characterized by a gradual and often irreversible decline in kidney function and may ultimately progress to end-stage kidney disease (ESKD) or kidney transplantation. Although established therapeutic strategies, including renin-angiotensin system (RAS) blockade and blood pressure control, can slow disease progression, many patients continue to experience progressive loss of kidney function, highlighting the need for additional nephroprotective therapies [2].

Sodium-glucose cotransporter-2 (SGLT-2) inhibitors, including empagliflozin, dapagliflozin, canagliflozin, and ertugliflozin, were initially developed as antihyperglycemic agents for the treatment of type 2 diabetes mellitus (T2DM). By inhibiting glucose reabsorption in the proximal renal tubule, these agents promote urinary glucose excretion and natriuresis. Large cardiovascular outcome trials subsequently demonstrated important renal benefits associated with SGLT-2 inhibition beyond their glucose-lowering effects [3,4]. These findings prompted further investigation into the broader nephroprotective effects of SGLT-2 inhibitors.

Subsequent landmark kidney outcome trials, including CREDENCE (Canagliflozin and Renal Events in Diabetes with Established Nephropathy Clinical Evaluation), DAPA-CKD (Dapagliflozin and Prevention of Adverse Outcomes in Chronic Kidney Disease), and EMPA-KIDNEY (Study of Heart and Kidney Protection with Empagliflozin), provided robust evidence that SGLT-2 inhibitors slow CKD progression and reduce the risk of kidney failure [5-7]. Importantly, DAPA-CKD and EMPA-KIDNEY extended these benefits to patients with CKD both with and without diabetes [6,7]. Collectively, these findings have transformed the therapeutic approach to CKD and established SGLT-2 inhibitors as a cornerstone of contemporary nephroprotective therapy.

The renoprotective effects of SGLT-2 inhibitors extend well beyond glucose lowering. Mechanistically, these agents restore tubuloglomerular feedback, resulting in afferent arteriolar vasoconstriction, reduced intraglomerular pressure, and attenuation of glomerular hyperfiltration [8]. Additional mechanisms include reduced renal oxygen consumption, decreased tubular workload, attenuation of inflammation and fibrosis, and modulation of oxidative stress and metabolic pathways [9]. Furthermore, SGLT-2 inhibitors improve systemic hemodynamics through modest reductions in blood pressure, body weight, and serum uric acid levels, thereby contributing to long-term preservation of kidney function [10].

Although numerous randomized controlled trials (RCTs) and meta-analyses have demonstrated the renoprotective efficacy of SGLT-2 inhibitors, several important questions remain. The magnitude of benefit may differ according to CKD etiology, baseline kidney function, albuminuria, and diabetes status [11]. In addition, the growing body of real-world evidence and mechanistic research warrants an updated synthesis to evaluate the consistency of available data, identify potential sources of heterogeneity, and highlight areas requiring further investigation.

Previous systematic reviews have evaluated selected aspects of SGLT-2 inhibitor therapy; however, many were limited by narrow inclusion criteria or did not incorporate recently published randomized trials and real-world observational studies [12,13]. Furthermore, uncertainty remains regarding the long-term renal safety of these agents and their efficacy in patients with advanced CKD or non-albuminuric disease.

The objective of this systematic review was to critically evaluate and synthesize the current evidence regarding the effects of SGLT-2 inhibitors on CKD progression in adults, irrespective of diabetes status, with particular emphasis on renal outcomes, including changes in estimated glomerular filtration rate (eGFR), progression to ESKD, and treatment-related adverse events.

## Review

Literature search strategy

This systematic review was conducted in accordance with the Preferred Reporting Items for Systematic Reviews and Meta-Analyses (PRISMA) 2020 statement to promote methodological transparency and reproducibility [14]. The review protocol was not prospectively registered in PROSPERO or another publicly accessible registry. Eligibility criteria were established using the PICOS (Population, Intervention, Comparator, Outcomes, and Study Design) framework. Eligible studies included adults (≥18 years) with CKD, irrespective of diabetes status, who received SGLT-2 inhibitor therapy, including dapagliflozin, empagliflozin, canagliflozin, ertugliflozin, or sotagliflozin. Randomized controlled trials, systematic reviews and meta-analyses, and prospective or retrospective observational cohort studies evaluating kidney outcomes were eligible for inclusion, whereas case reports, editorials, commentaries, and preclinical studies were excluded. Primary outcomes included CKD progression, changes in eGFR, doubling of serum creatinine, and progression to ESKD. Secondary outcomes included albuminuria, acute kidney injury, cardiovascular mortality, and all-cause mortality.

A systematic literature search was performed in PubMed/MEDLINE, Scopus, and Google Scholar from database inception through October 2025. The search strategy combined controlled vocabulary, where applicable, and free-text terms related to SGLT-2 inhibitors, chronic kidney disease, and kidney outcomes. Search terms included “sodium-glucose cotransporter-2 inhibitors,” “SGLT2 inhibitors,” “dapagliflozin,” “empagliflozin,” “canagliflozin,” “ertugliflozin,” “sotagliflozin,” “chronic kidney disease,” “CKD,” “renal impairment,” “kidney failure,” “nephropathy,” “eGFR,” “albuminuria,” “CKD progression,” and “renal outcomes.” Boolean operators (AND/OR) were used to combine the search concepts. The database-specific search strategies are provided in the Appendices. Two independent reviewers screened titles and abstracts and subsequently assessed potentially eligible full-text articles. Disagreements regarding study eligibility were resolved through discussion and consensus between the two reviewers. The study selection process is summarized in the PRISMA 2020 flow diagram (Figure 1). Owing to substantial clinical and methodological heterogeneity among the included studies, no de novo quantitative meta-analysis was performed, and the findings were synthesized narratively. As this review was based exclusively on previously published studies, institutional ethical approval and informed consent were not required.

**Figure 1:**
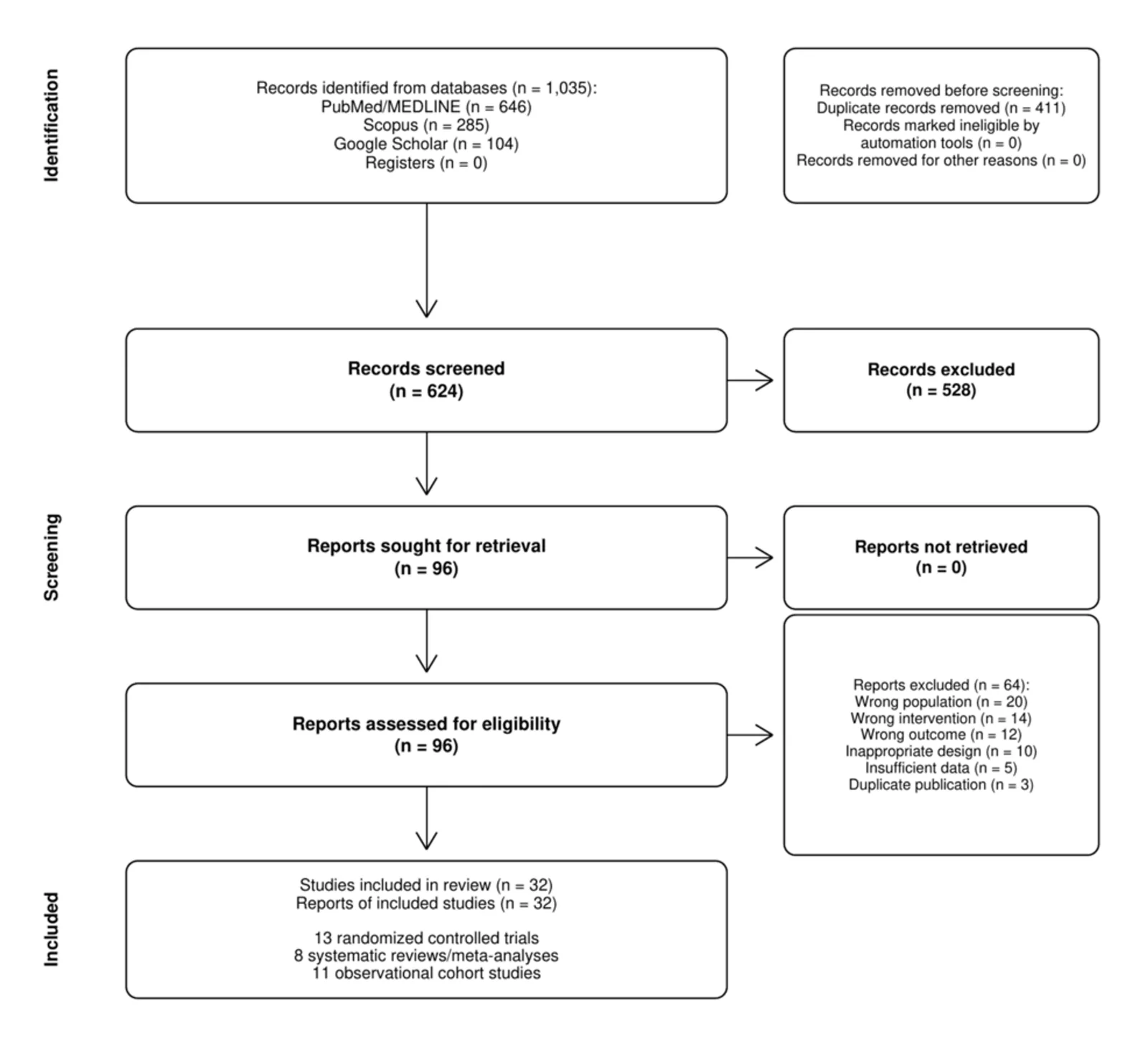
PRISMA 2020 flow diagram PRISMA: Preferred Reporting Items for Systematic Reviews and Meta-Analyses

Study selection and characteristics

A total of 32 studies met the inclusion criteria, comprising 13 randomized controlled trials, eight systematic reviews and meta-analyses, and 11 observational cohort studies. The included studies represented diverse patient populations, including individuals with T2DM, chronic kidney disease, heart failure, and varying degrees of kidney impairment. Study populations varied considerably in size, baseline kidney function, albuminuria status, and follow-up duration, reflecting the broad clinical spectrum in which SGLT-2 inhibitors have been evaluated. The characteristics and principal kidney-related findings of the included studies are summarized in Table 1.

**Table 1:** Characteristics of the 32 included studies AKI: acute kidney injury; ASCVD: atherosclerotic cardiovascular disease; CANVAS Program: Canagliflozin Cardiovascular Assessment Study; CKD: chronic kidney disease; CREDENCE: Canagliflozin and Renal Events in Diabetes With Established Nephropathy Clinical Evaluation; Cr: creatinine; CV: cardiovascular; CVD: cardiovascular disease; DAPA-CKD: Dapagliflozin and Prevention of Adverse Outcomes in Chronic Kidney Disease; DAPA-HF: Dapagliflozin and Prevention of Adverse Outcomes in Chronic Heart Failure; DECLARE-TIMI 58: Dapagliflozin Effect on Cardiovascular Events–Thrombolysis in Myocardial Infarction 58; DELIVER: Dapagliflozin Evaluation to Improve the Lives of Patients With Preserved Ejection Fraction Heart Failure; DPP-4: dipeptidyl peptidase-4; eGFR: estimated glomerular filtration rate; EMPA-REG OUTCOME: Empagliflozin Cardiovascular Outcome Event Trial in Type 2 Diabetes Mellitus Patients; EMPEROR-Preserved: Empagliflozin Outcome Trial in Patients With Chronic Heart Failure and a Preserved Ejection Fraction; EMPEROR-Reduced: Empagliflozin Outcome Trial in Patients With Chronic Heart Failure and a Reduced Ejection Fraction; ESKD: end-stage kidney disease; GLP-1 RA: glucagon-like peptide-1 receptor agonist; HF: heart failure; HFrEF: heart failure with reduced ejection fraction; LVEF: left ventricular ejection fraction; RAS: renin-angiotensin system; RCT: randomized controlled trial; SCORED: Sotagliflozin on Cardiovascular and Renal Events in Patients With Type 2 Diabetes and Moderate Renal Impairment; SGLT-2: sodium-glucose cotransporter-2; SOLOIST-WHF: Effect of Sotagliflozin on Cardiovascular Events in Patients With Diabetes and Recent Worsening Heart Failure; T2DM: type 2 diabetes mellitus; UACR: urinary albumin-to-creatinine ratio; VERTIS CV: Evaluation of Ertugliflozin Efficacy and Safety Cardiovascular Outcomes Trial; y: years

Study (year)	Design	Population	Sample	Intervention	Comparator	Follow-up	Renal outcome/principal finding
Randomized controlled trials (n = 13)							
Zinman et al. (2015), EMPA-REG OUTCOME [3]	RCT	T2DM with established CVD	7,020	Empagliflozin 10/25 mg	Placebo	3.1 y	Reduced incident or worsening nephropathy and major renal events.
Neal et al. (2017), CANVAS Program [4]	RCT	T2DM with high CV risk	10,142	Canagliflozin 100-300 mg	Placebo	2.4 y	Reduced progression of albuminuria and composite kidney outcomes.
Wiviott et al. (2019), DECLARE-TIMI 58 [15]	RCT	T2DM with ASCVD or multiple risk factors	17,160	Dapagliflozin 10 mg	Placebo	4.2 y	Reduced the prespecified renal composite outcome.
Cannon et al. (2020), VERTIS CV [16]	RCT	T2DM with established ASCVD	8,246	Ertugliflozin 5/15 mg	Placebo	3.5 y	Renal composite favored ertugliflozin, although not statistically significant in the primary hierarchy.
McMurray et al. (2019), DAPA-HF [17]	RCT	HFrEF with or without T2DM	4,744	Dapagliflozin 10 mg	Placebo	1.5 y	Slowed eGFR decline and reduced serious renal adverse events in secondary analyses.
Packer et al. (2020), EMPEROR-Reduced [18]	RCT	HFrEF with or without T2DM	3,730	Empagliflozin 10 mg	Placebo	1.3 y	Slowed annual eGFR decline and reduced a composite renal outcome.
Bhatt et al. (2021), SOLOIST-WHF [19]	RCT	T2DM after worsening heart failure	1,222	Sotagliflozin 200-400 mg	Placebo	0.8 y	Cardiorenal benefit observed; renal events were secondary outcomes.
Anker et al. (2021), EMPEROR-Preserved [20]	RCT	HF with LVEF >40%, with or without T2DM	5,988	Empagliflozin 10 mg	Placebo	2.2 y	Slowed eGFR decline; effect on strict kidney composite was less certain.
Solomon et al. (2022), DELIVER [21]	RCT	HF with mildly reduced/preserved EF	6,263	Dapagliflozin 10 mg	Placebo	2.3 y	Reduced HF outcomes and slowed kidney-function decline in secondary analyses.
Perkovic et al. (2019), CREDENCE [5]	RCT	T2DM with albuminuric CKD	4,401	Canagliflozin 100 mg	Placebo	2.6 y	30% lower risk of kidney failure, doubling of creatinine, or renal/CV death.
Heerspink et al. (2020), DAPA-CKD [6]	RCT	CKD with or without T2DM	4,304	Dapagliflozin 10 mg	Placebo	2.4 y	39% lower risk of sustained >=50% eGFR decline, ESKD, or renal/CV death.
Bhatt et al. (2021), SCORED [22]	RCT	T2DM with CKD	10,584	Sotagliflozin 200-400 mg	Placebo	1.3 y	Reduced cardiovascular events; kidney outcomes were directionally favorable.
Herrington et al. (2023), EMPA-KIDNEY [7]	RCT	CKD with or without T2DM	6,609	Empagliflozin 10 mg	Placebo	2.0 y	28% lower risk of kidney disease progression or cardiovascular death.
Systematic reviews and meta-analyses (n = 8)							
Toyama et al. (2019) [12]	Systematic review/meta-analysis	T2DM with CKD	RCT data	SGLT-2 inhibitors	Placebo/control	Varied	Reduced composite renal outcomes and attenuated annual eGFR decline.
Neuen et al. (2019) [13]	Systematic review/meta-analysis	T2DM across kidney-function strata	38,723	SGLT-2 inhibitors	Placebo	Varied	37% lower risk of dialysis, transplantation, or death from kidney disease.
Wang et al. (2019) [23]	Systematic review/meta-analysis	T2DM with or without CKD	Multiple RCTs	SGLT-2 inhibitors	Placebo	Varied	Renal protection was consistent across baseline kidney-disease status.
Li et al. (2021) [24]	Meta-analysis	CKD subgroups from RCTs	25,749 with eGFR <60	SGLT-2 inhibitors	Placebo	Varied	30% lower primary renal-outcome risk in patients with eGFR <60.
SMART-C Collaboration (2022) [25]	Collaborative meta-analysis	T2DM, HF, or CKD	90,413; 13 RCTs	SGLT-2 inhibitors	Placebo	0.8-4.2 y	37% lower kidney-disease progression risk, similar with and without diabetes.
Duo et al. (2023) [26]	Systematic review/meta-analysis	Adults receiving SGLT-2 inhibitors	Multiple RCTs	SGLT-2 inhibitors	Placebo/control	Varied	SGLT-2 inhibition slowed the rate of kidney-function decline.
Mavrakanas et al. (2023) [27]	Systematic review/meta-analysis	Patients with CKD	Multiple RCTs	SGLT-2 inhibitors	Placebo/control	Varied	Improved cardiovascular and renal outcomes and reduced AKI risk.
Forbes et al. (2023) [28]	Systematic review/meta-analysis of observational studies	Real-world T2DM populations	Multiple cohorts	SGLT-2 inhibitors	Other glucose-lowering drugs	Varied	Real-world evidence confirmed lower risk of adverse kidney outcomes.
Observational studies (n = 11)							
Heerspink et al. (2020), CVD-REAL 3 [11]	Multinational cohort	T2DM in routine practice	65,231 patients	SGLT-2 inhibitor initiation	Other glucose-lowering drugs	~1 y	Slower eGFR decline and lower risk of >=50% eGFR loss or ESKD.
Birkeland et al. (2021) [29]	Multinational cohort	T2DM without established CV/renal disease	Large multinational cohort	SGLT-2 inhibitors	DPP-4 inhibitors	Varied	Lower cardiorenal risk with SGLT-2 inhibitors.
Komuro et al. (2021) [30]	Japanese cohort	T2DM without established CV/renal disease	National real-world cohort	SGLT-2 inhibitors	Other glucose-lowering drugs	Varied	Lower risks of heart failure and CKD outcomes.
Idris et al. (2021) [31]	UK retrospective cohort	T2DM with/without prior CV or kidney disease	UK primary-care cohort	SGLT-2 inhibitors	DPP-4 inhibitors	Varied	Lower hospitalization for kidney disease, heart failure, and death.
Liu et al. (2022) [32]	Retrospective cohort	T2DM with diabetic kidney disease	>4,000	SGLT-2 inhibitors	Standard care/other agents	Varied	Lower CKD-progression risk (adjusted HR approximately 0.60).
Al-Muhaiteeb et al. (2024) [33]	Observational cohort	T2DM with and without renal impairment	Real-world cohort	SGLT-2 inhibitors	Non-use/other therapy	Long-term	Lower long-term mortality, including among patients with renal impairment.
Narasaki et al. (2024) [34]	Population-based Veterans cohort	US veterans with/without CKD	Large national cohort	SGLT-2 inhibitors	DPP-4 inhibitors/GLP-1 RAs	Varied	Favorable kidney and safety outcomes compared with alternative agents.
Song et al. (2024) [35]	Retrospective real-world study	T2DM	Real-world cohort	SGLT-2 inhibitors	Non-use/standard care	Varied	Better preservation of renal function during follow-up.
Chang et al. (2025) [36]	Nationwide observational cohort	T2DM	National database	SGLT-2 inhibitors	Other glucose-lowering drugs	Varied	Lower incidence of adverse renal outcomes with SGLT-2 inhibitors.
Huang et al. (2025) [37]	Retrospective cohort	T2DM with CKD stage 5	National/health-system cohort	SGLT-2 inhibitors	Non-use	Varied	Lower risk of renal failure and adverse outcomes in advanced CKD.
Hong et al. (2025) [38]	Nationwide cohort	Diabetes and hypertension	National cohort	SGLT-2 inhibitor and/or RAS inhibitor	Alternative treatment patterns	Varied	SGLT-2 inhibitor use, alone or with RAS blockade, was associated with lower ESKD/death risk.

Risk of bias assessment

Risk of bias was assessed using tools appropriate to the design of the included studies. Randomized controlled trials were evaluated using the revised Cochrane Risk of Bias tool (RoB 2), which assesses bias arising from the randomization process, deviations from intended interventions, missing outcome data, measurement of outcomes, and selection of the reported result [39]. Systematic reviews and meta-analyses were evaluated using the Risk of Bias in Systematic Reviews (ROBIS) tool, which assesses concerns related to study eligibility criteria, identification and selection of studies, data collection and study appraisal, and synthesis and interpretation of findings [40]. Observational cohort studies were assessed using the Risk Of Bias In Non-randomized Studies of Interventions (ROBINS-I) tool, which evaluates bias due to confounding, participant selection, classification of interventions, deviations from intended interventions, missing data, outcome measurement, and selection of reported results [41]. Overall judgments were categorized according to the criteria of each respective tool. The results of the risk-of-bias assessment are summarized in Table 2.

**Table 2:** Risk of bias assessment of included studies CANVAS, Canagliflozin Cardiovascular Assessment Study; CREDENCE, Canagliflozin and Renal Events in Diabetes With Established Nephropathy Clinical Evaluation; DAPA-CKD, Dapagliflozin and Prevention of Adverse Outcomes in Chronic Kidney Disease; DAPA-HF, Dapagliflozin and Prevention of Adverse Outcomes in Heart Failure; DECLARE-TIMI 58, Dapagliflozin Effect on Cardiovascular Events–Thrombolysis in Myocardial Infarction 58; DELIVER, Dapagliflozin Evaluation to Improve the Lives of Patients With Preserved Ejection Fraction Heart Failure; EMPA-KIDNEY, Study of Heart and Kidney Protection With Empagliflozin; EMPA-REG OUTCOME, Empagliflozin Cardiovascular Outcome Event Trial in Type 2 Diabetes Mellitus Patients; EMPEROR-Preserved, Empagliflozin Outcome Trial in Patients With Chronic Heart Failure With a Preserved Ejection Fraction; EMPEROR-Reduced, Empagliflozin Outcome Trial in Patients With Chronic Heart Failure and a Reduced Ejection Fraction; RCT, randomized controlled trial; ROBINS-I, Risk of Bias in Non-Randomized Studies of Interventions tool; ROBIS, Risk of Bias in Systematic Reviews tool; RoB 2, risk of bias tool; SCORED, Sotagliflozin on Cardiovascular and Renal Events in Patients With Type 2 Diabetes and Chronic Kidney Disease; SOLOIST-WHF, Sotagliflozin on Cardiovascular Events in Patients With Type 2 Diabetes and Recent Worsening Heart Failure; VERTIS CV, Evaluation of Ertugliflozin Efficacy and Safety Cardiovascular Outcomes Trial

Study	Design	Tool	Overall judgment	Principal consideration
Zinman et al., EMPA-REG OUTCOME [3]	RCT	RoB 2	Low risk	Randomized, double-blind, placebo-controlled; prespecified outcomes
Neal et al., CANVAS [4]	RCT	RoB 2	Low risk	Central randomization, masking and structured follow-up
Wiviott et al., DECLARE-TIMI 58 [15]	RCT	RoB 2	Low risk	Randomized, double-blind, placebo-controlled design
Cannon et al., VERTIS CV [16]	RCT	RoB 2	Low risk	Randomized, double-blind, placebo-controlled design
McMurray et al., DAPA-HF [17]	RCT	RoB 2	Low risk	Randomized, double-blind trial; kidney outcomes prespecified secondary analyses
Packer et al., EMPEROR-Reduced [18]	RCT	RoB 2	Low risk	Randomized, double-blind, placebo-controlled design
Bhatt et al., SOLOIST-WHF [19]	RCT	RoB 2	Some concerns	Early termination and modification of endpoint strategy
Anker et al., EMPEROR-Preserved [20]	RCT	RoB 2	Low risk	Randomized, double-blind, placebo-controlled design
Solomon et al., DELIVER [21]	RCT	RoB 2	Low risk	Randomized, double-blind, placebo-controlled design
Perkovic et al., CREDENCE [5]	RCT	RoB 2	Low risk	Randomized, double-blind trial with adjudicated kidney outcome
Heerspink et al., DAPA-CKD [6]	RCT	RoB 2	Low risk	Randomized, double-blind trial with prespecified kidney outcome
Bhatt et al., SCORED [22]	RCT	RoB 2	Some concerns	Trial ended early; primary endpoint changed and complete adjudication was not performed
Herrington et al., EMPA-KIDNEY [7]	RCT	RoB 2	Low risk	Randomized, double-blind, placebo-controlled kidney-outcome trial
Toyama et al. [12]	Systematic review/meta-analysis	ROBIS	Some concerns	Generally appropriate review methods, but limitations in prospective protocol/reporting
Neuen et al. [13]	Systematic review/meta-analysis	ROBIS	Low risk	Prespecified eligibility, systematic search, PROSPERO registration and appropriate synthesis
Wang et al. [23]	Systematic review/meta-analysis	ROBIS	Some concerns	Broad database search and study-level RoB assessment, but prospective protocol not clearly reported
Li et al. [24]	Systematic review/meta-analysis	ROBIS	Low risk	PROSPERO registration, broad search, independent extraction/RoB assessment and sensitivity analyses
SMART-C Collaboration [25]	Collaborative systematic review/meta-analysis	ROBIS	Low risk	Registered protocol, duplicate assessment/extraction and standardized outcome definitions
Duo et al. [26]	Systematic review/meta-analysis	ROBIS	Low risk	Prespecified/registered protocol, multiple databases, independent screening and study appraisal
Mavrakanas et al. [27]	Systematic review/meta-analysis	ROBIS	Some concerns	Prospective registration and duplicate appraisal, but literature search was primarily limited to MEDLINE
Forbes et al. [28]	Systematic review/meta-analysis	ROBIS	Low risk	Multiple databases, independent review, ROBINS-I appraisal and sensitivity analyses
Heerspink et al., CVD-REAL 3 [11]	Observational cohort	ROBINS-I	Moderate risk	Extensive propensity-score matching, but residual/unmeasured confounding remains possible
Birkeland et al. [29]	Observational cohort	ROBINS-I	Moderate risk	New-user design and propensity matching; residual confounding inherent to observational data
Komuro et al. [30]	Observational cohort	ROBINS-I	Moderate risk	Propensity-score matching with balanced groups; claims-based residual confounding possible
Idris et al. [31]	Retrospective cohort	ROBINS-I	Moderate risk	Propensity matching and new-user approach; residual confounding and outcome-code misclassification possible
Liu et al. [32]	Retrospective cohort	ROBINS-I	Moderate risk	Multivariable adjustment for major renal predictors; non-random treatment allocation remains
Al-Muhaiteeb et al. [33]	Observational cohort	ROBINS-I	Moderate risk	Propensity matching and multivariable adjustment; residual confounding remains possible
Narasaki et al. [34]	Population-based cohort	ROBINS-I	Moderate risk	Extensive adjustment, multiple imputation and propensity analyses; observational treatment allocation
Song et al. [35]	Retrospective single-center cohort	ROBINS-I	Serious risk	No concurrent untreated comparator; substantial potential for confounding and temporal bias
Chang et al. [36]	Nationwide observational cohort	ROBINS-I	Moderate risk	Propensity matching and balanced measured covariates; residual confounding remains possible
Huang et al. [37]	Retrospective nationwide cohort	ROBINS-I	Moderate risk	Propensity matching and competing-risk analyses; small treated CKD5 group and residual confounding
Hong et al. [38]	Nationwide cohort	ROBINS-I	Moderate risk	Large national dataset and adjusted analyses; confounding by indication remains possible

Evidence from randomized controlled trials

Evidence from randomized controlled trials consistently demonstrates that SGLT-2 inhibitors confer substantial renoprotective benefits across a broad spectrum of patients with T2DM, CKD, and heart failure. Early cardiovascular outcome trials, including EMPA-REG OUTCOME (Empagliflozin Cardiovascular Outcome Event Trial in Type 2 Diabetes Mellitus Patients; Zinman et al., 2015), CANVAS (Canagliflozin Cardiovascular Assessment Study; Neal et al., 2017), DECLARE-TIMI 58 (Dapagliflozin Effect on Cardiovascular Events-Thrombolysis in Myocardial Infarction 58; Wiviott et al., 2019), and VERTIS CV (Evaluation of Ertugliflozin Efficacy and Safety Cardiovascular Outcomes Trial; Cannon et al., 2020), provided early evidence of favorable renal effects of SGLT-2 inhibition, including reductions in albuminuria progression, slower deterioration of kidney function, and lower risks of composite renal outcomes [3,4,15,16]. Although these trials were primarily designed to evaluate cardiovascular outcomes, their renal findings provided important early evidence supporting the nephroprotective effects of SGLT-2 inhibition [3,4,15,16].

Subsequent heart failure trials, including DAPA-HF (Dapagliflozin and Prevention of Adverse Outcomes in Heart Failure; McMurray et al., 2019), EMPEROR-Reduced (Empagliflozin Outcome Trial in Patients With Chronic Heart Failure and a Reduced Ejection Fraction; Packer et al., 2020), SOLOIST-WHF (Sotagliflozin on Cardiovascular Events in Patients With Type 2 Diabetes and Recent Worsening Heart Failure; Bhatt et al., 2021), EMPEROR-Preserved (Empagliflozin Outcome Trial in Patients With Chronic Heart Failure With a Preserved Ejection Fraction; Anker et al., 2021), and DELIVER (Dapagliflozin Evaluation to Improve the Lives of Patients With Preserved Ejection Fraction Heart Failure; Solomon et al., 2022), further demonstrated the cardiorenal benefits of SGLT-2 inhibition in patients with heart failure, including favorable effects on the trajectory of kidney function [17-21]. These benefits were generally observed in patients with and without diabetes [17-21].

The dedicated kidney outcome trials provided the strongest evidence for renal protection. In the CREDENCE trial, canagliflozin reduced the risk of the primary composite renal outcome by 30% (hazard ratio (HR) 0.70; 95% confidence interval (CI), 0.59-0.82; p < 0.001) in patients with T2DM and CKD. Similarly, DAPA-CKD demonstrated a 39% reduction in the risk of sustained eGFR decline of ≥50%, progression to ESKD, or renal or cardiovascular death (HR 0.61; 95% CI, 0.51-0.72), while EMPA-KIDNEY reported a 28% reduction in the risk of kidney disease progression or cardiovascular death with empagliflozin (HR 0.72; 95% CI, 0.64-0.82). The SCORED (Sotagliflozin on Cardiovascular and Renal Events in Patients With Type 2 Diabetes and Moderate Renal Impairment) trial further contributed evidence regarding the cardiorenal effects of SGLT-2 inhibition in patients with T2DM and CKD [5-7,22].

Collectively, these randomized trials demonstrate that SGLT-2 inhibitors slow CKD progression, preserve kidney function, and reduce the risk of kidney failure across diverse patient populations, including individuals with and without diabetes, supporting their role as a cornerstone of contemporary nephroprotective therapy.

Evidence from meta-analyses

The renoprotective effects observed in individual randomized controlled trials have been further supported by multiple systematic reviews and meta-analyses. Collectively, the included meta-analyses demonstrated that SGLT-2 inhibitors reduce the risk of CKD progression and adverse kidney outcomes while preserving kidney function across diverse patient populations [12,13,23-28]. Importantly, these benefits were observed across a broad range of baseline kidney function and were maintained in individuals with and without diabetes [25].

Meta-analytic evidence further demonstrated that SGLT-2 inhibitor therapy significantly slowed the annual decline in eGFR, supporting preservation of kidney function compared with control therapy [26,27]. Importantly, subgroup analyses showed that the renal benefits were consistent irrespective of baseline diabetes status, with a similar reduction in kidney disease progression observed in patients without diabetes (RR 0.69; 95% CI, 0.57-0.82), supporting the concept that the nephroprotective effects of SGLT-2 inhibitors extend beyond glucose lowering [25].

Recent meta-analyses also confirmed a favorable renal safety profile of SGLT-2 inhibitors, including a reduced risk of acute kidney injury, while demonstrating consistent renoprotective effects across diverse CKD populations [25,27]. Furthermore, a systematic review and meta-analysis of real-world observational studies demonstrated favorable kidney outcomes associated with SGLT-2 inhibitor use, supporting the effectiveness and generalizability of SGLT-2 inhibitor therapy in routine clinical practice [28].

Real-world and observational evidence

The renal benefits of SGLT-2 inhibitors observed in randomized controlled trials have been further supported by evidence from real-world clinical practice. Observational studies, including multinational cohorts, nationwide database analyses, and retrospective cohort studies, demonstrated that SGLT-2 inhibitor therapy was associated with slower CKD progression, better preservation of kidney function, and lower risks of adverse kidney outcomes across diverse patient populations [29-38].

The multinational CVD-REAL 3 (Comparative Effectiveness of Cardiovascular and Renal Outcomes with Sodium-Glucose Cotransporter-2 Inhibitors; Heerspink et al., 2020) study provided large-scale real-world evidence that initiation of SGLT-2 inhibitors was associated with a slower decline in eGFR and a lower risk of major adverse kidney outcomes compared with other glucose-lowering therapies [11]. Similar findings were subsequently reported in multinational and national observational studies by Birkeland et al. and Komuro et al., demonstrating lower risks of cardiorenal and kidney outcomes among patients receiving SGLT-2 inhibitors in routine clinical practice [29,30]. Likewise, Idris et al. reported favorable cardiorenal outcomes with SGLT-2 inhibitors compared with dipeptidyl peptidase-4 inhibitors, including lower risks of hospitalization for heart failure, kidney-related outcomes, and all-cause mortality, irrespective of prior cardiovascular or kidney disease [31].

Further evidence from more recent observational studies reinforced these findings. Liu et al. demonstrated approximately a 40% reduction in CKD progression among patients with diabetic kidney disease treated with SGLT-2 inhibitors (adjusted HR 0.60; 95% CI, 0.49-0.74) [32]. Similarly, Narasaki et al. reported favorable kidney and safety outcomes in a large U.S. Veterans cohort, while Al-Muhaiteeb et al. observed improved long-term survival in patients with type 2 diabetes mellitus receiving SGLT-2 inhibitors [33,34]. Additional nationwide and retrospective cohort studies by Song et al., Chang et al., Huang et al., and Hong et al. further demonstrated favorable kidney outcomes associated with SGLT-2 inhibitor use across patients with varying degrees of kidney impairment, including advanced CKD [35-38].

Collectively, these observational studies complement the evidence from randomized controlled trials by demonstrating that the favorable kidney outcomes associated with SGLT-2 inhibitor therapy are also observed under routine clinical practice conditions. The consistency of findings across different healthcare systems, study designs, and patient populations strengthens the external validity of randomized trial evidence and supports the integration of SGLT-2 inhibitors into standard nephroprotective therapy for patients with CKD [29-38].

Safety profile

Overall, SGLT-2 inhibitors demonstrated a favorable safety profile across randomized controlled trials, systematic reviews, meta-analyses, and real-world observational studies. The incidence of serious adverse events was generally comparable to placebo or standard therapy, supporting the use of SGLT-2 inhibitors in patients with chronic kidney disease, including those without diabetes [6,7,12,13,25,27].

Meta-analytic evidence demonstrated that SGLT-2 inhibitor therapy was associated with a significant reduction in the risk of acute kidney injury among patients with CKD (RR 0.82; 95% CI, 0.72-0.93) [27]. This protective effect is consistent with broader trial-level evidence demonstrating favorable kidney outcomes in patients both with and without diabetes [25]. Although volume depletion is a recognized adverse effect of SGLT-2 inhibition, appropriate clinical monitoring is warranted, particularly in patients at increased risk of hypovolemia.

Across major randomized controlled trials, genital mycotic infections occurred more frequently with SGLT-2 inhibitors than with placebo, consistent with their glucosuric mechanism of action. These events were generally mild and infrequently resulted in treatment discontinuation [3-7,15-22]. Severe hypoglycemia was not increased, while ketoacidosis remained uncommon across the major trials. Importantly, the overall safety profile remained favorable in CKD populations with and without diabetes [6,7,25]. Meta-analytic evidence further demonstrated a reduced risk of acute kidney injury among patients with CKD receiving SGLT-2 inhibitors (RR 0.82; 95% CI, 0.72-0.93) [27].

Real-world observational studies further supported the favorable safety profile of SGLT-2 inhibitors under routine clinical practice conditions. These studies reported favorable kidney and safety outcomes across diverse patient populations, including individuals with varying degrees of kidney impairment and multiple comorbidities [29-38].

Collectively, the available evidence indicates that SGLT-2 inhibitors are generally well tolerated, with a favorable benefit-risk profile that supports their long-term use as cornerstone nephroprotective therapy in patients with CKD.

Discussion

The findings of this systematic review provide comprehensive and up-to-date evidence that SGLT-2 inhibitors substantially slow the progression of CKD across a broad spectrum of patient populations, irrespective of diabetes status. By synthesizing evidence from 32 studies, including 13 randomized controlled trials, eight systematic reviews and meta-analyses, and 11 observational cohort studies, this review demonstrates consistent reductions in CKD progression, preservation of kidney function, and decreased risks of ESKD and major adverse renal outcomes. Across the included evidence, SGLT-2 inhibitors reduced the relative risk of CKD progression by approximately 23-30% while significantly slowing the decline in eGFR compared with placebo or standard therapy.

The renoprotective effects were consistently observed across all currently evaluated SGLT-2 inhibitors, including dapagliflozin, empagliflozin, canagliflozin, ertugliflozin, and sotagliflozin, and were evident in patients with diabetic and non-diabetic CKD, heart failure, and high cardiovascular risk. The consistency of benefit across diverse clinical settings suggests that the nephroprotective effects of SGLT-2 inhibitors extend beyond glucose lowering and are mediated through complementary hemodynamic, metabolic, and anti-inflammatory mechanisms that preserve long-term kidney function.

The present findings are supported by a robust body of randomized evidence. Early cardiovascular outcome trials, including EMPA-REG OUTCOME, CANVAS, DECLARE-TIMI 58, and VERTIS CV, provided early evidence of clinically meaningful renal benefits among patients with type 2 diabetes mellitus [3,4,15,16]. Subsequently, dedicated kidney outcome trials, particularly CREDENCE, DAPA-CKD, SCORED, and EMPA-KIDNEY, established SGLT-2 inhibition as an effective nephroprotective strategy by demonstrating substantial reductions in adverse kidney outcomes across diverse CKD populations. Importantly, DAPA-CKD and EMPA-KIDNEY extended this evidence to patients with CKD both with and without diabetes [5-7,22]. In parallel, heart failure trials, including DAPA-HF, EMPEROR-Reduced, SOLOIST-WHF, EMPEROR-Preserved, and DELIVER, further demonstrated the cardiorenal benefits of SGLT-2 inhibition in patients with heart failure, with generally favorable effects on kidney-function trajectories [17-21].

The randomized evidence is further reinforced by the included systematic reviews and meta-analyses. Collectively, these studies demonstrated significant reductions in CKD progression and preservation of kidney function with SGLT-2 inhibitor therapy across diverse patient populations [12,13,23-27]. Importantly, pooled analyses demonstrated that kidney benefits were maintained in individuals without diabetes, supporting the concept that the nephroprotective effects of SGLT-2 inhibitors extend beyond glycemic control [25]. Furthermore, a systematic review and meta-analysis of real-world observational studies, together with individual observational cohort studies, reported favorable kidney outcomes associated with SGLT-2 inhibitor use in routine clinical practice, supporting the external validity and generalizability of randomized trial findings [28-38].

Clinical implications

These findings have important implications for contemporary clinical practice. The consistent efficacy observed across diabetic and non-diabetic CKD populations supports current guideline recommendations for the use of SGLT-2 inhibitors in appropriately selected patients with CKD, irrespective of diabetes status [42]. Their favorable safety profile and complementary mechanisms of action support the integration of SGLT-2 inhibitors with established nephroprotective strategies, including RAS blockade, to preserve kidney function and delay progression to kidney failure [42].

Beyond nephroprotection, SGLT-2 inhibitors provide important cardiovascular benefits, particularly through reductions in hospitalization for heart failure, reinforcing their role within the cardiorenal continuum. Evidence from EMPA-KIDNEY further demonstrates substantial kidney benefits in patients with advanced CKD, including those with an eGFR as low as 20 mL/min/1.73 m² [7]. Recent observational studies have complemented randomized trial evidence by demonstrating favorable cardiorenal outcomes in routine clinical practice among patients with varying degrees of kidney impairment [33,36,38].

Collectively, the available evidence establishes SGLT-2 inhibitors as a cornerstone of contemporary CKD management. Their ability to preserve kidney function, reduce the risk of kidney failure, improve cardiovascular outcomes, and maintain a favorable safety profile supports their incorporation into standard therapy for appropriately selected patients with CKD. Future research should further define their long-term efficacy in advanced and non-albuminuric CKD and evaluate combination treatment strategies to optimize individualized nephroprotective care.

Remaining evidence gaps in advanced CKD, including patients with very low eGFR, non-albuminuric disease, and other underrepresented populations, are discussed further in the Strengths and Limitations and Future Research Directions sections.

Strengths and limitations

This systematic review has several notable strengths. It was conducted in accordance with the PRISMA 2020 guidelines, promoting methodological transparency and reproducibility. A comprehensive literature search of PubMed/MEDLINE, Scopus, and Google Scholar identified 32 eligible studies, including randomized controlled trials, systematic reviews and meta-analyses, and real-world observational cohort studies published between 2015 and 2025. By integrating evidence from multiple study designs, this review provides a comprehensive synthesis of the efficacy and safety of SGLT-2 inhibitors across diverse CKD populations, including patients with and without diabetes. Furthermore, study selection was performed independently by two reviewers, minimizing the potential for selection bias and strengthening the methodological rigor of the review. Risk of bias was systematically assessed using design-specific validated tools, allowing methodological limitations of the included evidence to be considered when interpreting the findings.

Nevertheless, several limitations should be acknowledged. First, considerable heterogeneity existed among the included studies with respect to CKD etiology, baseline kidney function, albuminuria, study populations, follow-up duration, and definitions of kidney outcomes, limiting direct quantitative comparisons across studies. Second, although the review incorporated evidence from randomized controlled trials, systematic reviews and meta-analyses, and observational cohort studies, no de novo quantitative meta-analysis was performed because of substantial clinical and methodological heterogeneity among the included studies. Third, several landmark randomized trials were industry-sponsored, which should be considered when interpreting the available evidence despite their rigorous methodology. In addition, the risk-of-bias assessment identified methodological concerns in some systematic reviews and observational studies, particularly those related to residual confounding and limitations inherent to non-randomized study designs, which should be considered when interpreting the overall findings. Fourth, patients with advanced CKD, particularly those with an eGFR<20 mL/min/1.73 m², kidney transplant recipients, and individuals with non-albuminuric CKD were underrepresented in the available evidence, limiting the generalizability of the findings to these populations. Finally, follow-up duration varied considerably across studies, and evidence regarding the long-term durability of nephroprotective effects and safety of SGLT-2 inhibitor therapy remains comparatively limited.

Future research directions

Although substantial evidence supports the efficacy and safety of SGLT-2 inhibitors in CKD, several important knowledge gaps remain. Future randomized controlled trials should further evaluate these agents in populations that remain underrepresented in the available evidence, including individuals with advanced CKD, particularly those with an eGFR<20 mL/min/1.73 m², patients with non-albuminuric CKD, kidney transplant recipients, and those with rare or less common causes of CKD. Additional studies with extended follow-up are also needed to establish the long-term durability of kidney protection and safety of SGLT-2 inhibitor therapy.

Further mechanistic investigations should clarify the non-glycemic pathways underlying SGLT-2 inhibitor-mediated renoprotection, including effects on renal hemodynamics, tubuloglomerular feedback, mitochondrial function, inflammation, oxidative stress, fibrosis, and metabolic remodeling. A better understanding of these mechanisms may facilitate more precise patient selection and contribute to the development of individualized nephroprotective strategies.

Future research should also investigate combination treatment strategies. In particular, studies evaluating SGLT-2 inhibitors in combination with non-steroidal mineralocorticoid receptor antagonists, such as finerenone, glucagon-like peptide-1 receptor agonists, and other emerging nephroprotective therapies are warranted to determine whether additional cardiorenal benefits can be achieved while maintaining an acceptable safety profile.

Finally, long-term real-world observational studies and pharmacovigilance registries are warranted to evaluate uncommon or delayed adverse events, treatment adherence, cost-effectiveness, and clinical outcomes across diverse healthcare settings. Such studies may complement randomized trial evidence and provide valuable information regarding the long-term safety and effectiveness of SGLT-2 inhibitors in routine clinical practice.

Addressing these research priorities may further refine patient selection, optimize therapeutic strategies, and improve kidney and cardiovascular outcomes in patients with CKD.

## Conclusions

This systematic review demonstrates that sodium-glucose cotransporter-2 (SGLT-2) inhibitors provide substantial renoprotective benefits across a broad spectrum of patients with chronic kidney disease (CKD). By synthesizing evidence from 32 studies, including randomized controlled trials, systematic reviews and meta-analyses, and real-world observational cohort studies, the available evidence indicates that SGLT-2 inhibitors reduce the risk of CKD progression, end-stage kidney disease (ESKD), and major adverse kidney outcomes while slowing the decline in estimated glomerular filtration rate (eGFR). Importantly, these benefits extend beyond glycemic control and are observed in patients both with and without diabetes.

The available evidence further supports complementary hemodynamic, metabolic, and anti-inflammatory mechanisms underlying SGLT-2 inhibitor-mediated nephroprotection, together with a favorable safety profile across diverse clinical settings. The consistency of findings across randomized trials and real-world observational studies supports the incorporation of SGLT-2 inhibitors into contemporary CKD management alongside renin-angiotensin system blockade and other evidence-based nephroprotective therapies. Although further research is needed in underrepresented populations, including patients with advanced and non-albuminuric CKD, the current evidence supports SGLT-2 inhibitors as a cornerstone of contemporary nephroprotective therapy, with the potential to improve kidney and cardiovascular outcomes and delay progression to kidney replacement therapy.

## Appendices

**Table 3:** Database-specific search strategies Search period: From database inception through October 2025. No restrictions based on diabetes status were applied.

Database	Search strategy
PubMed/MEDLINE	("sodium-glucose cotransporter 2 inhibitors"[MeSH Terms] OR "SGLT2 inhibitors" OR dapagliflozin OR empagliflozin OR canagliflozin OR ertugliflozin OR sotagliflozin) AND ("chronic kidney disease"[MeSH Terms] OR "chronic kidney disease" OR CKD OR "renal impairment" OR "kidney failure" OR nephropathy) AND (progression OR decline OR eGFR OR albuminuria OR "renal outcomes" OR "kidney outcomes" OR "renal protection")
Scopus	TITLE-ABS-KEY(("sodium-glucose cotransporter 2 inhibitor*" OR "SGLT2 inhibitor*" OR dapagliflozin OR empagliflozin OR canagliflozin OR ertugliflozin OR sotagliflozin) AND ("chronic kidney disease" OR CKD OR "renal impairment" OR "kidney failure" OR nephropathy) AND (progression OR decline OR eGFR OR albuminuria OR "renal outcome*" OR "kidney outcome*" OR "renal protection"))
Google Scholar	"SGLT2 inhibitors" AND ("chronic kidney disease" OR CKD) AND (progression OR eGFR OR albuminuria OR "kidney outcomes" OR "renal outcomes")
